# Bis(tetra­phenyl­arsonium) di-μ-hy­droxido-bis­[(nitrilo­triacetato)­cobalt(III)] octa­hydrate

**DOI:** 10.1107/S160053681003895X

**Published:** 2010-10-13

**Authors:** Hendrik G. Visser, Jack K. Clegg

**Affiliations:** a Department of Chemistry, University of the Free State, PO Box 339, Bloemfontein 9300, South Africa; b Department of Chemistry, University of Cambridge, Lensfield Rd, Cambridge CB2 1EW, England

## Abstract

In the title compound, (C_24_H_20_As)_2_[Co_2_(C_6_H_6_NO_6_)_2_(OH)_2_]·8H_2_O, the Co^III^ atom in the binuclear centrosymmetric anion is octa­hedrally surrounded by one N atom and three O atoms of the tetra­dentate nitrilo­triacetate ligand and two μ-hydroxide ligands. The crystal packing is controlled by C—H⋯O and O—H⋯O hydrogen-bonding inter­actions. The crystal employed in this study proved to be a two-component twin around (0
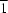
1).

## Related literature

For synthetic background, related structures and kinetics, see: Mori *et al.* (1958 ▶); Visser *et al.* (1997 ▶, 1999 ▶, 2001 ▶, 2002 ▶, 2003 ▶). For twinning, which was resolved using the program *ROTAX*, see: Cooper *et al.* (2002 ▶).
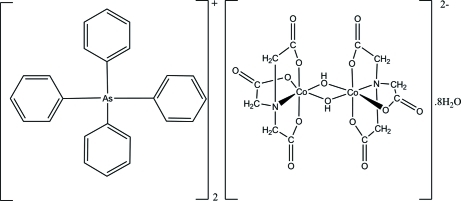


## Experimental

### 

#### Crystal data

(C_24_H_20_As)_2_[Co_2_(C_6_H_6_NO_6_)_2_(OH)_2_]·8H_2_O
*M* *_r_* = 1438.88
Triclinic, 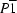

*a* = 7.328 (5) Å
*b* = 14.491 (5) Å
*c* = 16.564 (5) Å
α = 113.555 (5)°
β = 97.040 (5)°
γ = 101.152 (5)°
*V* = 1542.4 (13) Å^3^
*Z* = 1
Mo *K*α radiation
μ = 1.68 mm^−1^
*T* = 100 K
0.44 × 0.19 × 0.03 mm

#### Data collection

Bruker X8 APEXII 4K KappaCCD diffractometer
Absorption correction: multi-scan (*SADABS*; Bruker, 2004 ▶) *T*_min_ = 0.687, *T*_max_ = 0.950
23209 measured reflections
23223 independent reflections
20726 reflections with *I* > 2σ(*I*)
*R*_int_ = 0.030

#### Refinement

*R*[*F* ^2^ > 2σ(*F* ^2^)] = 0.030
*wR*(*F* ^2^) = 0.098
*S* = 0.93
23223 reflections
430 parameters
10 restraints
H atoms treated by a mixture of independent and constrained refinement
Δρ_max_ = 0.41 e Å^−3^
Δρ_min_ = −0.36 e Å^−3^

### 

Data collection: *APEX2* (Bruker, 2005 ▶); cell refinement: *SAINT-Plus* (Bruker, 2004 ▶); data reduction: *SAINT-Plus* and *XPREP* (Bruker, 2004 ▶); program(s) used to solve structure: *SIR97* (Altomare *et al.*, 1999 ▶); program(s) used to refine structure: *SHELXL97* (Sheldrick, 2008 ▶); molecular graphics: *DIAMOND* (Brandenburg & Putz, 2005 ▶); software used to prepare material for publication: *WinGX* (Farrugia, 1999 ▶).

## Supplementary Material

Crystal structure: contains datablocks global, I. DOI: 10.1107/S160053681003895X/kj2157sup1.cif

Structure factors: contains datablocks I. DOI: 10.1107/S160053681003895X/kj2157Isup2.hkl

Additional supplementary materials:  crystallographic information; 3D view; checkCIF report

## Figures and Tables

**Table 1:** Selected bond lengths (Å)

Co1—O1	1.9027 (11)
Co1—O3	1.8903 (11)
Co1—O5	1.8806 (11)
Co1—O7	1.8915 (11)
Co1—O7^i^	1.8994 (11)
Co1—N1	1.9312 (13)

Symmetry code: (i) 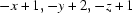.

**Table 2:** Hydrogen-bond geometry (Å, °)

*D*—H⋯*A*	*D*—H	H⋯*A*	*D*⋯*A*	*D*—H⋯*A*
C4—H4*A*⋯O4^ii^	0.97	2.49	3.239 (2)	134
C13—H13⋯O6^iii^	0.93	2.39	3.1207 (19)	135
C14—H14⋯O6^iv^	0.93	2.55	3.146 (3)	123
C15—H15⋯O5^iv^	0.93	2.58	3.313 (3)	136
C23—H23⋯O7	0.93	2.56	3.4034 (19)	150
C52—H2⋯O9^v^	0.93	2.53	3.386 (2)	153
O7—H7⋯O4^vi^	0.77 (2)	2.00 (2)	2.740 (2)	162 (2)
O8—H8*A*⋯O10^vii^	0.82 (2)	1.94 (2)	2.758 (2)	178 (2)
O8—H8*B*⋯O2	0.78 (2)	1.96 (2)	2.7329 (17)	171 (2)
O9—H9*A*⋯O8	0.80 (2)	2.25 (2)	3.045 (2)	170 (2)
O9—H9*B*⋯O8^viii^	0.82 (2)	1.92 (2)	2.7290 (19)	169 (3)
O10—H10*B*⋯O11	0.76 (2)	2.09 (2)	2.8483 (18)	174 (2)
O10—H10*C*⋯O9^ix^	0.82 (2)	1.95 (2)	2.771 (2)	175 (2)
O11—H11*A*⋯O4^iii^	0.76 (2)	2.22 (2)	2.9542 (16)	164 (2)
O11—H11*B*⋯O2^ix^	0.89 (2)	2.02 (2)	2.905 (2)	172 (2)

Symmetry codes: (ii) 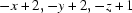; (iii) 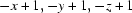; (iv) 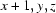; (v) 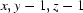; (vi) 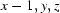; (vii) 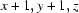; (viii) 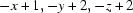; (ix) 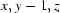.

**Fig. 1.**
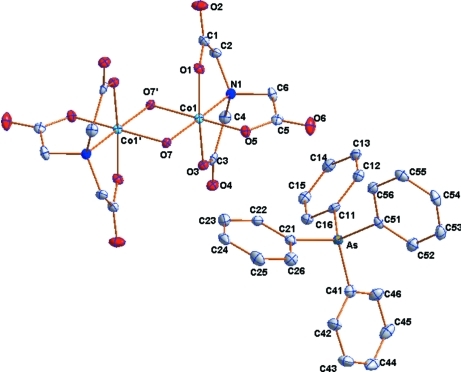
View of the title compund (50% probability displacement ellipsoids). Hydrogen atoms and solvent water molecules omitted for clarity.

## supplementary crystallographic information

### Comment

The title complex, (AsC_24_H_2_0)_2_[Co(C_6_H_6_N)(µ-OH)]_2_.8(H_2_O), forms a part of an ongoing investigation of the structural and kinetic behaviour of Co^III^-nitrilotriacetato compounds (Visser *et al.*, (1997, 1999, 2001, 2002, 2003). The Co^III^ atom is octahedrally surrounded by one N atom and three O atoms of the tetradentate nitrilotriacetato ligand and two µ-hydroxo ligands (Fig. 1). The Co–O bond distances which vary between 1.8806 (11) and 1.9027 (11) Å and the Co–N1 distance of 1.9312 (13) Å fall well within the normal range (Table 1). The octahedron around the Co atom is only slighlty distorted. The crystal packing is controlled by C—H–O and O—H–O hydrogen bonding interactions (Table 2), creating a three dimensional network.

### Experimental

The title compound was prepared similar to the method described by Mori *et al.* (1958). CoCl_2_.6H_2_O (5 g, 21 mmol) and nitrilotriacetic acid (4 g, 21 mmol) was added to a KHCO_3_ solution (25 cm^3^, 2.5 *M*). H_2_O_2_ (1 ml, 30%) was added to this solution and the mixture was placed on an ice bath. After 5 h a purple precipitate separated out. The precipitate was filtered and washed several times with cold water. The product was then redissolved in hot water after which an excess of tetraphenylarsonium was added. Purple crystals of the title compound were obtained after several days.

### Refinement

The methyl and aromatic H atoms were placed in geometrically idealized positions (C—H = 0.93–0.96 Å) and constrained to ride on their parent atoms, with *U*_iso_(H) = 1.5*U*_eq_(C) and 1.2*U*_eq_(C), respectively. The hydrogen atoms of the aqua solvent molecules were obtained from the difference Fourier map. Despite appearing (at least visually) to be single, the crystal employed in this study proved to be a two-component twin around (0 -1 1). Twinning which was resolved using the programs ROTAX (Cooper *et al.*, 2002) and Make HKLF5 (Farrugia, 1999) with a final minor component of 0.0712 (4).

### Crystal data

**Table:**

(C_24_H_20_As)_2_[Co_2_(C_6_H_6_NO_6_)_2_(OH)_2_]·8H_2_O	*Z* = 1
*M**_r_* = 1438.88	*F*(000) = 740
Triclinic, *P*1	*D*_x_ = 1.549 Mg m^−^^3^
Hall symbol: -P 1	Mo *K*α radiation, λ = 0.71073 Å
*a* = 7.328 (5) Å	Cell parameters from 9875 reflections
*b* = 14.491 (5) Å	θ = 2.5–28.3°
*c* = 16.564 (5) Å	µ = 1.68 mm^−^^1^
α = 113.555 (5)°	*T* = 100 K
β = 97.040 (5)°	Plate, purple
γ = 101.152 (5)°	0.44 × 0.19 × 0.03 mm
*V* = 1542.4 (13) Å^3^	

### Data collection

**Table:**

Bruker X8 APEXII 4K KappaCCD diffractometer	23223 independent reflections
Radiation source: sealed tube	20726 reflections with *I* > 2σ(*I*)
graphite	*R*_int_ = 0.030
Detector resolution: 0 pixels mm^-1^	θ_max_ = 25.0°, θ_min_ = 1.6°
phi and ω scans	*h* = −8→8
Absorption correction: multi-scan (*SADABS*; Bruker, 2004)	*k* = −17→17
*T*_min_ = 0.687, *T*_max_ = 0.950	*l* = −19→19
23209 measured reflections	

### Refinement

**Table:**

Refinement on *F*^2^	Primary atom site location: structure-invariant direct methods
Least-squares matrix: full	Secondary atom site location: difference Fourier map
*R*[*F*^2^ > 2σ(*F*^2^)] = 0.030	Hydrogen site location: inferred from neighbouring sites
*wR*(*F*^2^) = 0.098	H atoms treated by a mixture of independent and constrained refinement
*S* = 0.93	*w* = 1/[σ^2^(*F*_o_^2^) + (0.0699*P*)^2^ + 0.5465*P*] where *P* = (*F*_o_^2^ + 2*F*_c_^2^)/3
23223 reflections	(Δ/σ)_max_ = 0.001
430 parameters	Δρ_max_ = 0.41 e Å^−^^3^
10 restraints	Δρ_min_ = −0.35 e Å^−^^3^

### Special details

**Table:**

Experimental. The crystal was coated in Exxon Paratone N hydrocarbon oil and mounted on a thin mohair fibre attached to a copper pin. Upon mounting on the diffractometer, the crystal was quenched to 100(K) under a cold nitrogen gas stream supplied by an Oxford Cryosystems Cryostream and data were collected at this temperature.
Geometry. All e.s.d.'s (except the e.s.d. in the dihedral angle between two l.s. planes) are estimated using the full covariance matrix. The cell e.s.d.'s are taken into account individually in the estimation of e.s.d.'s in distances, angles and torsion angles; correlations between e.s.d.'s in cell parameters are only used when they are defined by crystal symmetry. An approximate (isotropic) treatment of cell e.s.d.'s is used for estimating e.s.d.'s involving l.s. planes.
Refinement. Refinement of *F*^2^ against ALL reflections. The weighted *R*-factor *wR* and goodness of fit *S* are based on *F*^2^, conventional *R*-factors *R* are based on *F*, with *F* set to zero for negative *F*^2^. The threshold expression of *F*^2^ > σ(*F*^2^) is used only for calculating *R*-factors(gt) *etc*. and is not relevant to the choice of reflections for refinement. *R*-factors based on *F*^2^ are statistically about twice as large as those based on *F*, and *R*- factors based on ALL data will be even larger.

### Fractional atomic coordinates and isotropic or equivalent isotropic displacement parameters (Å^2^)

**Table:**

	*x*	*y*	*z*	*U*_iso_*/*U*_eq_	
As	0.39743 (2)	0.447716 (10)	0.212481 (9)	0.01293 (5)	
Co1	0.52032 (3)	0.909913 (13)	0.511641 (11)	0.00933 (6)	
O5	0.37750 (13)	0.76948 (7)	0.46088 (6)	0.0136 (2)	
O6	0.37392 (15)	0.61846 (7)	0.46771 (7)	0.0226 (2)	
O3	0.67319 (13)	0.87442 (7)	0.42572 (6)	0.0118 (2)	
O7	0.35509 (14)	0.94527 (7)	0.43838 (6)	0.0103 (2)	
C25	−0.0013 (2)	0.57427 (12)	0.12621 (10)	0.0239 (4)	
H25	−0.1144	0.5486	0.0827	0.029*	
C22	0.3389 (2)	0.65222 (11)	0.25432 (9)	0.0183 (3)	
H22	0.4537	0.6783	0.2969	0.022*	
C24	0.0660 (2)	0.68072 (12)	0.18135 (10)	0.0249 (4)	
H24	−0.0029	0.7261	0.1754	0.030*	
N1	0.69776 (16)	0.87921 (8)	0.58731 (7)	0.0111 (2)	
C44	0.6324 (2)	0.27716 (12)	−0.04076 (10)	0.0270 (4)	
H44	0.6776	0.2413	−0.0912	0.032*	
C54	−0.0652 (2)	0.19678 (11)	0.24101 (10)	0.0223 (4)	
H4	−0.1589	0.1472	0.2459	0.027*	
C13	0.7874 (2)	0.53345 (10)	0.45190 (9)	0.0159 (3)	
H13	0.7972	0.5165	0.5005	0.019*	
C11	0.6130 (2)	0.51328 (10)	0.31153 (9)	0.0141 (3)	
C16	0.7629 (2)	0.58563 (10)	0.30779 (9)	0.0171 (3)	
H36	0.7538	0.6028	0.2593	0.021*	
C12	0.6230 (2)	0.48623 (10)	0.38328 (9)	0.0145 (3)	
H12	0.5222	0.4378	0.3854	0.017*	
C3	0.8420 (2)	0.87705 (10)	0.45975 (9)	0.0114 (3)	
C55	−0.0350 (2)	0.30323 (11)	0.29762 (9)	0.0189 (3)	
H5	−0.1083	0.3244	0.3403	0.023*	
C6	0.6159 (2)	0.76932 (10)	0.57216 (9)	0.0168 (3)	
H6B	0.5795	0.7698	0.6267	0.020*	
H6A	0.7129	0.7317	0.5603	0.020*	
C23	0.2356 (2)	0.71896 (12)	0.24507 (10)	0.0241 (4)	
H23	0.2805	0.7903	0.2820	0.029*	
C5	0.4429 (2)	0.71259 (11)	0.49372 (9)	0.0150 (3)	
C4	0.8820 (2)	0.89285 (11)	0.55782 (9)	0.0136 (3)	
H4B	0.9468	0.8425	0.5631	0.016*	
H4A	0.9641	0.9624	0.5962	0.016*	
C51	0.2135 (2)	0.34422 (10)	0.22566 (9)	0.0141 (3)	
C21	0.2698 (2)	0.54575 (11)	0.19945 (9)	0.0155 (3)	
C46	0.6374 (2)	0.33354 (11)	0.11640 (10)	0.0209 (3)	
H46	0.6868	0.3365	0.1721	0.025*	
C14	0.9360 (2)	0.60521 (11)	0.44813 (9)	0.0169 (3)	
H14	1.0451	0.6362	0.4945	0.020*	
C53	0.0440 (2)	0.16579 (11)	0.17818 (10)	0.0229 (4)	
H3	0.0234	0.0951	0.1410	0.028*	
C45	0.7061 (2)	0.28103 (11)	0.04135 (10)	0.0258 (4)	
H45	0.8017	0.2485	0.0465	0.031*	
C15	0.9256 (2)	0.63202 (10)	0.37639 (9)	0.0170 (3)	
H15	1.0266	0.6805	0.3745	0.020*	
C52	0.1852 (2)	0.23883 (11)	0.16938 (9)	0.0190 (3)	
H2	0.2587	0.2175	0.1269	0.023*	
C56	0.1045 (2)	0.37675 (11)	0.28960 (9)	0.0163 (3)	
H21	0.1252	0.4475	0.3268	0.020*	
C41	0.4944 (2)	0.38168 (10)	0.10790 (9)	0.0165 (3)	
C42	0.4214 (2)	0.37872 (12)	0.02556 (10)	0.0243 (4)	
H42	0.3265	0.4115	0.0201	0.029*	
C26	0.0983 (2)	0.50630 (11)	0.13542 (10)	0.0195 (3)	
H27	0.0516	0.4348	0.0993	0.023*	
C43	0.4924 (3)	0.32580 (12)	−0.04900 (10)	0.0322 (4)	
H43	0.4448	0.3233	−0.1047	0.039*	
O1	0.38813 (13)	0.94103 (7)	0.60717 (6)	0.0128 (2)	
O4	0.97100 (13)	0.86981 (7)	0.41750 (6)	0.0142 (2)	
O2	0.45349 (15)	1.00672 (8)	0.75721 (6)	0.0219 (2)	
C1	0.4993 (2)	0.96884 (10)	0.68350 (9)	0.0144 (3)	
C2	0.7004 (2)	0.95505 (11)	0.68087 (9)	0.0143 (3)	
H2A	0.7902	1.0216	0.6967	0.017*	
H2B	0.7395	0.9288	0.7238	0.017*	
O8	0.68108 (17)	1.03630 (9)	0.91382 (8)	0.0247 (3)	
O11	0.17288 (17)	0.12264 (9)	0.75373 (7)	0.0268 (3)	
O9	0.33579 (19)	1.08138 (10)	0.99202 (9)	0.0304 (3)	
O10	0.04230 (18)	0.13286 (10)	0.91136 (8)	0.0327 (3)	
H9B	0.315 (4)	1.0439 (17)	1.0176 (15)	0.079 (9)*	
H8B	0.616 (3)	1.0211 (15)	0.8671 (13)	0.056 (5)*	
H8A	0.789 (3)	1.0632 (15)	0.9127 (13)	0.056 (5)*	
H9A	0.430 (3)	1.0771 (13)	0.9721 (12)	0.035 (4)*	
H10B	0.069 (3)	0.1294 (14)	0.8674 (12)	0.047 (4)*	
H11A	0.157 (3)	0.1295 (14)	0.7106 (12)	0.044 (4)*	
H11B	0.265 (3)	0.0912 (13)	0.7531 (12)	0.044 (4)*	
H7	0.254 (3)	0.9294 (13)	0.4445 (11)	0.035 (4)*	
H10C	0.126 (3)	0.1179 (14)	0.9378 (12)	0.047 (4)*	

### Atomic displacement parameters (Å^2^)

**Table:**

	*U*^11^	*U*^22^	*U*^33^	*U*^12^	*U*^13^	*U*^23^
As	0.01200 (8)	0.01345 (9)	0.01245 (8)	0.00238 (6)	0.00164 (6)	0.00552 (6)
Co1	0.00692 (10)	0.01049 (10)	0.01102 (10)	0.00240 (8)	0.00233 (8)	0.00496 (8)
O5	0.0106 (5)	0.0127 (5)	0.0169 (5)	0.0017 (4)	0.0024 (4)	0.0068 (4)
O6	0.0148 (6)	0.0148 (5)	0.0392 (6)	0.0025 (5)	0.0063 (5)	0.0133 (5)
O3	0.0095 (5)	0.0139 (5)	0.0123 (5)	0.0044 (4)	0.0034 (4)	0.0051 (4)
O7	0.0070 (5)	0.0105 (5)	0.0128 (5)	0.0014 (4)	0.0021 (4)	0.0049 (4)
C25	0.0205 (9)	0.0330 (9)	0.0250 (8)	0.0095 (8)	0.0061 (7)	0.0180 (7)
C22	0.0254 (9)	0.0190 (8)	0.0117 (7)	0.0051 (7)	0.0077 (7)	0.0071 (6)
C24	0.0349 (10)	0.0310 (9)	0.0272 (9)	0.0213 (8)	0.0195 (8)	0.0217 (8)
N1	0.0099 (6)	0.0112 (6)	0.0125 (6)	0.0030 (5)	0.0024 (5)	0.0055 (5)
C44	0.0320 (10)	0.0193 (8)	0.0241 (9)	0.0003 (7)	0.0169 (8)	0.0040 (7)
C54	0.0172 (8)	0.0223 (8)	0.0296 (9)	−0.0005 (7)	0.0019 (7)	0.0170 (7)
C13	0.0173 (8)	0.0187 (8)	0.0146 (7)	0.0092 (7)	0.0038 (6)	0.0081 (6)
C11	0.0154 (8)	0.0130 (7)	0.0114 (7)	0.0048 (6)	0.0026 (6)	0.0025 (6)
C16	0.0192 (8)	0.0176 (8)	0.0157 (7)	0.0060 (7)	0.0055 (6)	0.0073 (6)
C12	0.0121 (7)	0.0133 (7)	0.0180 (7)	0.0038 (6)	0.0040 (6)	0.0061 (6)
C3	0.0112 (7)	0.0058 (7)	0.0145 (7)	0.0004 (6)	0.0017 (6)	0.0029 (6)
C55	0.0159 (8)	0.0254 (8)	0.0187 (8)	0.0059 (7)	0.0042 (6)	0.0126 (7)
C6	0.0175 (8)	0.0154 (7)	0.0226 (8)	0.0044 (6)	0.0054 (7)	0.0130 (6)
C23	0.0397 (11)	0.0175 (8)	0.0191 (8)	0.0104 (8)	0.0139 (8)	0.0087 (7)
C5	0.0123 (8)	0.0153 (8)	0.0208 (8)	0.0049 (6)	0.0104 (6)	0.0086 (6)
C4	0.0095 (7)	0.0154 (7)	0.0174 (7)	0.0045 (6)	0.0034 (6)	0.0079 (6)
C51	0.0119 (7)	0.0157 (7)	0.0143 (7)	0.0012 (6)	−0.0009 (6)	0.0084 (6)
C21	0.0170 (8)	0.0178 (7)	0.0153 (7)	0.0051 (6)	0.0065 (6)	0.0097 (6)
C46	0.0171 (8)	0.0211 (8)	0.0179 (8)	0.0033 (7)	0.0000 (7)	0.0040 (6)
C14	0.0126 (8)	0.0173 (8)	0.0159 (7)	0.0052 (6)	0.0007 (6)	0.0025 (6)
C53	0.0237 (9)	0.0139 (8)	0.0257 (8)	0.0011 (7)	0.0013 (7)	0.0061 (7)
C45	0.0191 (9)	0.0192 (8)	0.0321 (9)	0.0040 (7)	0.0078 (7)	0.0042 (7)
C15	0.0145 (8)	0.0152 (7)	0.0211 (8)	0.0043 (6)	0.0079 (6)	0.0066 (6)
C52	0.0184 (8)	0.0189 (8)	0.0190 (8)	0.0069 (7)	0.0052 (7)	0.0064 (6)
C56	0.0182 (8)	0.0139 (7)	0.0144 (7)	0.0053 (6)	0.0014 (6)	0.0040 (6)
C41	0.0154 (8)	0.0140 (7)	0.0164 (7)	−0.0001 (6)	0.0046 (6)	0.0046 (6)
C42	0.0298 (10)	0.0277 (9)	0.0204 (8)	0.0116 (8)	0.0080 (7)	0.0131 (7)
C26	0.0191 (8)	0.0184 (8)	0.0212 (8)	0.0043 (7)	0.0049 (7)	0.0091 (6)
C43	0.0478 (12)	0.0314 (9)	0.0189 (8)	0.0078 (9)	0.0119 (8)	0.0125 (7)
O1	0.0102 (5)	0.0159 (5)	0.0142 (5)	0.0039 (4)	0.0042 (4)	0.0078 (4)
O4	0.0097 (5)	0.0163 (5)	0.0160 (5)	0.0031 (4)	0.0051 (4)	0.0058 (4)
O2	0.0205 (6)	0.0339 (6)	0.0144 (5)	0.0131 (5)	0.0085 (5)	0.0096 (5)
C1	0.0147 (8)	0.0144 (7)	0.0164 (8)	0.0031 (6)	0.0041 (6)	0.0092 (6)
C2	0.0142 (8)	0.0177 (7)	0.0119 (7)	0.0054 (6)	0.0034 (6)	0.0065 (6)
O8	0.0244 (7)	0.0317 (7)	0.0205 (6)	0.0069 (6)	0.0048 (5)	0.0142 (5)
O11	0.0276 (7)	0.0400 (7)	0.0196 (6)	0.0191 (6)	0.0096 (5)	0.0141 (6)
O9	0.0297 (7)	0.0379 (7)	0.0403 (7)	0.0168 (6)	0.0195 (6)	0.0267 (6)
O10	0.0264 (7)	0.0510 (8)	0.0320 (7)	0.0129 (6)	0.0107 (6)	0.0267 (7)

### Geometric parameters (Å, °)

**Table:**

As—C11	1.9049 (15)	C3—O3	1.2816 (18)
As—C21	1.9102 (15)	C3—C4	1.5288 (19)
As—C51	1.9145 (15)	C55—C56	1.387 (2)
As—C41	1.9134 (15)	C55—H5	0.9300
Co1—O5	1.8806 (11)	C6—C5	1.520 (2)
Co1—O1	1.9027 (11)	C6—H6B	0.9700
Co1—O3	1.8903 (11)	C6—H6A	0.9700
Co1—O5	1.8806 (11)	C23—H23	0.9300
Co1—O3	1.8903 (11)	C5—O6	1.2295 (16)
Co1—O7	1.8915 (11)	C5—O5	1.2855 (17)
Co1—O7	1.8915 (11)	C4—H4B	0.9700
Co1—O7^i^	1.8994 (11)	C4—H4A	0.9700
Co1—N1	1.9312 (13)	C51—C56	1.391 (2)
Co1—Co1^i^	2.8516 (10)	C51—C52	1.3916 (19)
O5—C5	1.2855 (17)	C21—C26	1.394 (2)
O6—C5	1.2295 (16)	C46—C41	1.389 (2)
O3—C3	1.2816 (18)	C46—C45	1.387 (2)
O7—Co1^i^	1.8994 (11)	C46—H46	0.9300
O7—H7	0.765 (18)	C14—C15	1.3881 (19)
C25—C26	1.377 (2)	C14—H14	0.9300
C25—C24	1.388 (2)	C53—C52	1.396 (2)
C25—H25	0.9300	C53—H3	0.9300
C22—C23	1.381 (2)	C45—H45	0.9300
C22—C21	1.388 (2)	C15—H15	0.9300
C22—H22	0.9300	C52—H2	0.9300
C24—C23	1.382 (2)	C56—H21	0.9300
C24—H24	0.9300	C41—C42	1.383 (2)
N1—C4	1.4956 (19)	C42—C43	1.392 (2)
N1—C6	1.4943 (17)	C42—H42	0.9300
N1—C2	1.4941 (17)	C26—H27	0.9300
C44—C43	1.375 (3)	C43—H43	0.9300
C44—C45	1.375 (2)	O1—C1	1.2799 (16)
C44—H44	0.9300	O2—C1	1.2415 (17)
C54—C53	1.376 (2)	C1—O2	1.2415 (17)
C54—C55	1.403 (2)	C1—C2	1.528 (2)
C54—H4	0.9300	C2—H2A	0.9700
C13—C14	1.379 (2)	C2—H2B	0.9700
C13—C12	1.391 (2)	O8—H8B	0.780 (19)
C13—H13	0.9300	O8—H8A	0.82 (2)
C11—C12	1.3906 (19)	O11—H11A	0.758 (17)
C11—C16	1.390 (2)	O11—H11B	0.886 (19)
C16—C15	1.383 (2)	O9—H9B	0.82 (2)
C16—H36	0.9300	O9—H9A	0.800 (19)
C12—H12	0.9300	O10—H10B	0.762 (18)
C3—O4	1.2396 (18)	O10—H10C	0.822 (18)
C3—O4	1.2396 (18)		
			
C11—As—C21	112.01 (6)	O4—C3—O3	122.95 (13)
C11—As—C51	111.91 (6)	O4—C3—O3	122.95 (13)
C21—As—C51	107.22 (7)	O4—C3—O3	122.95 (13)
C11—As—C41	105.75 (7)	O4—C3—C4	120.09 (12)
C21—As—C41	110.79 (6)	O4—C3—C4	120.09 (12)
C51—As—C41	109.18 (6)	O3—C3—C4	116.94 (12)
O5—Co1—O3	89.83 (5)	O3—C3—C4	116.94 (12)
O5—Co1—O3	89.83 (5)	C56—C55—C54	119.66 (15)
O5—Co1—O3	89.83 (5)	C56—C55—H5	120.2
O5—Co1—O3	89.83 (5)	C54—C55—H5	120.2
O5—Co1—O7	93.60 (5)	N1—C6—C5	112.41 (11)
O5—Co1—O7	93.60 (5)	N1—C6—H6B	109.1
O3—Co1—O7	92.04 (5)	C5—C6—H6B	109.1
O3—Co1—O7	92.04 (5)	N1—C6—H6A	109.1
O5—Co1—O7	93.60 (5)	C5—C6—H6A	109.1
O5—Co1—O7	93.60 (5)	H6B—C6—H6A	107.9
O3—Co1—O7	92.04 (5)	C24—C23—C22	120.60 (14)
O3—Co1—O7	92.04 (5)	C24—C23—H23	119.7
O7—Co1—O7	0.00 (5)	C22—C23—H23	119.7
O5—Co1—O7^i^	175.18 (4)	O6—C5—O5	125.03 (13)
O5—Co1—O7^i^	175.18 (4)	O6—C5—O5	125.03 (13)
O3—Co1—O7^i^	93.03 (5)	O6—C5—O5	125.03 (13)
O3—Co1—O7^i^	93.03 (5)	O6—C5—O5	125.03 (13)
O7—Co1—O7^i^	82.43 (5)	O6—C5—C6	119.13 (12)
O7—Co1—O7^i^	82.43 (5)	O6—C5—C6	119.13 (12)
O5—Co1—O1	89.72 (5)	O5—C5—C6	115.82 (12)
O5—Co1—O1	89.72 (5)	O5—C5—C6	115.82 (12)
O3—Co1—O1	172.72 (4)	N1—C4—C3	109.35 (11)
O3—Co1—O1	172.72 (4)	N1—C4—H4B	109.8
O7—Co1—O1	95.25 (6)	C3—C4—H4B	109.8
O7—Co1—O1	95.25 (6)	N1—C4—H4A	109.8
O7^i^—Co1—O1	87.93 (4)	C3—C4—H4A	109.8
O5—Co1—N1	88.78 (5)	H4B—C4—H4A	108.3
O5—Co1—N1	88.78 (5)	C56—C51—C52	121.13 (13)
O3—Co1—N1	87.07 (6)	C56—C51—As	118.76 (10)
O3—Co1—N1	87.07 (6)	C52—C51—As	120.06 (11)
O7—Co1—N1	177.45 (4)	C22—C21—C26	120.58 (14)
O7—Co1—N1	177.45 (4)	C22—C21—As	121.78 (11)
O7^i^—Co1—N1	95.23 (5)	C26—C21—As	117.57 (11)
O1—Co1—N1	85.65 (6)	C41—C46—C45	119.64 (14)
O5—Co1—Co1^i^	134.86 (4)	C41—C46—H46	120.2
O5—Co1—Co1^i^	134.86 (4)	C45—C46—H46	120.2
O3—Co1—Co1^i^	93.37 (3)	C13—C14—C15	121.14 (13)
O3—Co1—Co1^i^	93.37 (3)	C13—C14—H14	119.4
O7—Co1—Co1^i^	41.32 (3)	C15—C14—H14	119.4
O7—Co1—Co1^i^	41.32 (3)	C54—C53—C52	121.04 (14)
O7^i^—Co1—Co1^i^	41.11 (4)	C54—C53—H3	119.5
O1—Co1—Co1^i^	92.10 (3)	C52—C53—H3	119.5
N1—Co1—Co1^i^	136.33 (3)	C44—C45—C46	119.77 (16)
C5—O5—Co1	115.09 (9)	C44—C45—H45	120.1
C3—O3—Co1	113.20 (9)	C46—C45—H45	120.1
Co1—O7—Co1^i^	97.57 (5)	C16—C15—C14	119.09 (14)
Co1—O7—H7	108.2 (13)	C16—C15—H15	120.5
Co1^i^—O7—H7	117.9 (13)	C14—C15—H15	120.5
C26—C25—C24	120.49 (15)	C53—C52—C51	118.51 (15)
C26—C25—H25	119.8	C53—C52—H2	120.7
C24—C25—H25	119.8	C51—C52—H2	120.7
C23—C22—C21	119.29 (14)	C55—C56—C51	119.68 (13)
C23—C22—H22	120.4	C55—C56—H21	120.2
C21—C22—H22	120.4	C51—C56—H21	120.2
C23—C24—C25	119.74 (14)	C46—C41—C42	120.64 (14)
C23—C24—H24	120.1	C46—C41—As	117.79 (11)
C25—C24—H24	120.1	C42—C41—As	121.54 (12)
C4—N1—C6	112.32 (10)	C41—C42—C43	118.91 (16)
C4—N1—C2	115.56 (11)	C41—C42—H42	120.5
C6—N1—C2	111.17 (10)	C43—C42—H42	120.5
C4—N1—Co1	106.22 (8)	C25—C26—C21	119.29 (14)
C6—N1—Co1	107.09 (8)	C25—C26—H27	120.4
C2—N1—Co1	103.62 (9)	C21—C26—H27	120.4
C43—C44—C45	120.61 (15)	C44—C43—C42	120.42 (15)
C43—C44—H44	119.7	C44—C43—H43	119.8
C45—C44—H44	119.7	C42—C43—H43	119.8
C53—C54—C55	119.99 (14)	C1—O1—Co1	111.85 (10)
C53—C54—H4	120.0	O2—C1—O1	123.92 (14)
C55—C54—H4	120.0	O2—C1—O1	123.92 (14)
C14—C13—C12	120.32 (13)	O2—C1—C2	119.79 (12)
C14—C13—H13	119.8	O2—C1—C2	119.79 (12)
C12—C13—H13	119.8	O1—C1—C2	116.28 (12)
C12—C11—C16	121.26 (13)	N1—C2—C1	108.05 (11)
C12—C11—As	120.89 (11)	N1—C2—H2A	110.1
C16—C11—As	117.79 (10)	C1—C2—H2A	110.1
C15—C16—C11	119.81 (13)	N1—C2—H2B	110.1
C15—C16—H36	120.1	C1—C2—H2B	110.1
C11—C16—H36	120.1	H2A—C2—H2B	108.4
C13—C12—C11	118.38 (13)	H8B—O8—H8A	107 (2)
C13—C12—H12	120.8	H11A—O11—H11B	108 (2)
C11—C12—H12	120.8	H9B—O9—H9A	112 (2)
O4—C3—O3	122.95 (13)	H10B—O10—H10C	107 (2)
			
O3—Co1—O5—C5	88.39 (10)	N1—C6—C5—O5	−9.19 (18)
O3—Co1—O5—C5	88.39 (10)	N1—C6—C5—O5	−9.19 (18)
O7—Co1—O5—C5	−179.58 (10)	C6—N1—C4—C3	91.91 (12)
O7—Co1—O5—C5	−179.58 (10)	C2—N1—C4—C3	−139.11 (11)
O1—Co1—O5—C5	−84.34 (10)	Co1—N1—C4—C3	−24.85 (12)
N1—Co1—O5—C5	1.32 (10)	O4—C3—C4—N1	−169.55 (11)
Co1^i^—Co1—O5—C5	−177.02 (8)	O4—C3—C4—N1	−169.55 (11)
O5—Co1—O3—C3	−107.97 (9)	O3—C3—C4—N1	12.06 (16)
O5—Co1—O3—C3	−107.97 (9)	O3—C3—C4—N1	12.06 (16)
O7—Co1—O3—C3	158.44 (9)	C11—As—C51—C56	72.54 (12)
O7—Co1—O3—C3	158.44 (9)	C21—As—C51—C56	−50.66 (12)
O7^i^—Co1—O3—C3	75.91 (9)	C41—As—C51—C56	−170.74 (11)
N1—Co1—O3—C3	−19.18 (9)	C11—As—C51—C52	−110.07 (12)
Co1^i^—Co1—O3—C3	117.09 (8)	C21—As—C51—C52	126.73 (11)
O5—Co1—O7—Co1^i^	177.26 (4)	C41—As—C51—C52	6.65 (13)
O5—Co1—O7—Co1^i^	177.26 (4)	C23—C22—C21—C26	0.2 (2)
O3—Co1—O7—Co1^i^	−92.78 (5)	C23—C22—C21—As	−176.62 (11)
O3—Co1—O7—Co1^i^	−92.78 (5)	C11—As—C21—C22	3.51 (14)
O1—Co1—O7—Co1^i^	87.20 (5)	C51—As—C21—C22	126.65 (12)
O5—Co1—N1—C4	114.14 (8)	C41—As—C21—C22	−114.30 (12)
O5—Co1—N1—C4	114.14 (8)	C11—As—C21—C26	−173.39 (11)
O3—Co1—N1—C4	24.25 (8)	C51—As—C21—C26	−50.25 (13)
O3—Co1—N1—C4	24.25 (8)	C41—As—C21—C26	68.80 (13)
O7^i^—Co1—N1—C4	−68.53 (8)	C55—C54—C53—C52	−0.2 (2)
O1—Co1—N1—C4	−156.04 (8)	C43—C44—C45—C46	0.7 (2)
Co1^i^—Co1—N1—C4	−67.56 (9)	C41—C46—C45—C44	0.1 (2)
O5—Co1—N1—C6	−6.07 (8)	C56—C51—C52—C53	0.2 (2)
O5—Co1—N1—C6	−6.07 (8)	As—C51—C52—C53	−177.17 (11)
O3—Co1—N1—C6	−95.97 (9)	C54—C55—C56—C51	−0.1 (2)
O3—Co1—N1—C6	−95.97 (9)	C52—C51—C56—C55	−0.1 (2)
O7^i^—Co1—N1—C6	171.26 (8)	As—C51—C56—C55	177.30 (10)
O1—Co1—N1—C6	83.74 (9)	C45—C46—C41—C42	−0.7 (2)
Co1^i^—Co1—N1—C6	172.23 (7)	C45—C46—C41—As	177.33 (11)
O5—Co1—N1—C2	−123.66 (9)	C11—As—C41—C46	39.24 (12)
O5—Co1—N1—C2	−123.66 (9)	C21—As—C41—C46	160.81 (11)
O3—Co1—N1—C2	146.44 (8)	C51—As—C41—C46	−81.33 (12)
O3—Co1—N1—C2	146.44 (8)	C11—As—C41—C42	−142.74 (12)
O7^i^—Co1—N1—C2	53.67 (9)	C21—As—C41—C42	−21.17 (13)
O1—Co1—N1—C2	−33.85 (8)	C51—As—C41—C42	96.69 (13)
Co1^i^—Co1—N1—C2	54.64 (10)	C46—C41—C42—C43	0.6 (2)
C21—As—C11—C12	119.23 (12)	As—C41—C42—C43	−177.37 (12)
C51—As—C11—C12	−1.21 (14)	C24—C25—C26—C21	−1.4 (2)
C41—As—C11—C12	−119.99 (12)	C22—C21—C26—C25	0.8 (2)
C21—As—C11—C16	−63.40 (13)	As—C21—C26—C25	177.78 (11)
C51—As—C11—C16	176.16 (11)	C45—C44—C43—C42	−0.8 (2)
C41—As—C11—C16	57.38 (13)	C41—C42—C43—C44	0.1 (2)
As—C11—C16—C15	−177.37 (11)	O5—Co1—O1—C1	114.55 (9)
As—C11—C12—C13	177.29 (10)	O5—Co1—O1—C1	114.55 (9)
Co1—O3—C3—O4	−170.26 (10)	O7—Co1—O1—C1	−151.86 (9)
Co1—O3—C3—O4	−170.26 (10)	O7—Co1—O1—C1	−151.86 (9)
Co1—O3—C3—C4	8.08 (14)	O7^i^—Co1—O1—C1	−69.66 (9)
C53—C54—C55—C56	0.3 (2)	N1—Co1—O1—C1	25.75 (9)
C4—N1—C6—C5	−106.93 (13)	Co1^i^—Co1—O1—C1	−110.57 (8)
C2—N1—C6—C5	121.84 (13)	Co1—O1—C1—O2	169.64 (11)
Co1—N1—C6—C5	9.30 (13)	Co1—O1—C1—O2	169.64 (11)
C25—C24—C23—C22	0.1 (2)	Co1—O1—C1—C2	−9.27 (14)
C21—C22—C23—C24	−0.6 (2)	C4—N1—C2—C1	151.43 (11)
Co1—O5—C5—O6	−177.51 (11)	C6—N1—C2—C1	−79.03 (13)
Co1—O5—C5—O6	−177.51 (11)	Co1—N1—C2—C1	35.68 (12)
Co1—O5—C5—C6	4.07 (15)	O2—C1—C2—N1	162.19 (12)
N1—C6—C5—O6	172.29 (12)	O2—C1—C2—N1	162.19 (12)
N1—C6—C5—O6	172.29 (12)	O1—C1—C2—N1	−18.86 (16)

Symmetry codes:  (i) −*x*+1, −*y*+2, −*z*+1.

### Hydrogen-bond geometry (Å, °)

**Table:**

*D*—H···*A*	*D*—H	H···*A*	*D*···*A*	*D*—H···*A*
C4—H4A···O4^ii^	0.97	2.49	3.239 (2)	134
C13—H13···O6^iii^	0.93	2.39	3.1207 (19)	135
C14—H14···O6^iv^	0.93	2.55	3.146 (3)	123
C15—H15···O5^iv^	0.93	2.58	3.313 (3)	136
C23—H23···O7	0.93	2.56	3.4034 (19)	150
C52—H2···O9^v^	0.93	2.53	3.386 (2)	153
O7—H7···O4^vi^	0.77 (2)	2.00 (2)	2.740 (2)	162 (2)
O8—H8A···O10^vii^	0.82 (2)	1.94 (2)	2.758 (2)	178 (2)
O8—H8B···O2	0.78 (2)	1.96 (2)	2.7329 (17)	171 (2)
O9—H9A···O8	0.80 (2)	2.25 (2)	3.045 (2)	170 (2)
O9—H9B···O8^viii^	0.82 (2)	1.92 (2)	2.7290 (19)	169 (3)
O10—H10B···O11	0.76 (2)	2.09 (2)	2.8483 (18)	174 (2)
O10—H10C···O9^ix^	0.82 (2)	1.95 (2)	2.771 (2)	175 (2)
O11—H11A···O4^iii^	0.76 (2)	2.22 (2)	2.9542 (16)	164 (2)
O11—H11B···O2^ix^	0.89 (2)	2.02 (2)	2.905 (2)	172 (2)

Symmetry codes: (ii) −*x*+2, −*y*+2, −*z*+1; (iii) −*x*+1, −*y*+1, −*z*+1; (iv) *x*+1, *y*, *z*;  (v) *x*, *y*−1, *z*−1; (vi) *x*−1, *y*, *z*; (vii) *x*+1, *y*+1, *z*; (viii) −*x*+1, −*y*+2, −*z*+2; (ix) *x*, *y*−1, *z*.
